# Machine learning prediction of sepsis in paralytic ileus using interpretable clinical models

**DOI:** 10.3389/fcimb.2026.1705126

**Published:** 2026-06-02

**Authors:** Qingzhou Song, Xuanlin Wu, Firooz Ahmad Taheri, Linghou Meng, Wentao Wang, Xianwei Mo

**Affiliations:** 1 Division of Colorectal & Anal Surgery, Department of Gastrointestinal Surgery, Guangxi Medical University Cancer Hospital, Nanning, Guangxi Zhuang Autonomous Region, China; 2 Department of Breast Surgery, Guangxi Medical University Cancer Hospital, Nanning, Guangxi Zhuang Autonomous Region, China

**Keywords:** machine learning, MIMIC-IV, paralytic ileus, predictive model, sepsis

## Abstract

**Background:**

Paralytic ileus (PI) is a common complication in critically ill patients, and the development of sepsis significantly worsens prognosis. Early identification of sepsis risk in PI patients remains a clinical challenge.

**Methods:**

Adult PI patients were identified from the MIMIC-IV database (2008–2022) and randomly split into a training cohort (70%) and an internal validation cohort (30%). An independent external cohort was obtained from Guangxi Medical University Cancer Hospital. Predictors were extracted within the first 12 hours after admission. Feature selection was performed using LASSO regression and the Boruta algorithm combined with clinical knowledge. Seven machine learning models were evaluated. Model performance was assessed by area under the receiver operating characteristic curve (AUC), calibration, and decision curve analysis (DCA). SHAP was applied for interpretability.

**Results:**

A total of 579 PI patients were included, of whom 29.78% developed new-onset sepsis. Seven predictors were retained: pneumonia, red cell distribution width (RDW), heart failure, blood urea nitrogen (BUN), atrial fibrillation, serum chloride, and white blood cell count. Logistic regression demonstrated stable performance and was selected as the final model, achieving an AUC of 0.687 in internal validation and 0.715 in external validation. Calibration and DCA indicated good agreement and consistent clinical net benefit. SHAP analysis identified pneumonia and RDW as the most influential predictors.

**Conclusion:**

An early, interpretable logistic regression model based on readily available clinical variables can effectively predict new-onset sepsis in PI patients and may support timely risk stratification and preventive intervention.

## 1 Introduction

Paralytic ileus (PI) is a non-mechanical form of intestinal obstruction arising from impaired gastrointestinal motility. It occurs frequently in critically ill patients and those undergoing abdominal surgery (Reichert et al., 2024). The etiology of PI is multifactorial, encompassing surgical trauma, severe infection, electrolyte imbalance, and systemic inflammatory responses. Clinically, PI is associated with prolonged hospitalization, increased complications, and higher healthcare costs (Sommer et al., 2021). Among its severe sequelae, sepsis poses a particular challenge in both diagnosis and management (Carlson and Dark, 2010). Sepsis, defined as life-threatening organ dysfunction caused by a dysregulated host response to infection, is a major driver of mortality in PI (Singer et al., 2016). Disruption of the intestinal barrier, bacterial translocation, immune dysfunction, and repeated invasive procedures such as surgery, mechanical ventilation, or catheterization collectively heighten the risk of secondary infection and sepsis (Doudakmanis et al., 2021; Charitos et al., 2025). Once sepsis develops, mortality rises sharply, underscoring the urgent need for early recognition of high-risk PI patients (Rudd et al., 2020).

Although the Surviving Sepsis Campaign guidelines emphasize early detection and timely intervention, accurate diagnosis of sepsis in PI remains difficult (Rhodes et al., 2017). Non-specific manifestations of PI—such as abdominal distension, reduced bowel sounds, and gastrointestinal dysmotility—often overlap with the systemic features of sepsis, obscuring its early signals (van Bree et al., 2014). Moreover, widely used risk stratification tools, including the Sequential Organ Failure Assessment (SOFA) and quick SOFA (qSOFA) scores, have limited sensitivity and specificity in this population and remain insufficiently validated in PI (Raith et al., 2017; Zhang Y. et al., 2020).

Recent advances in machine learning (ML) have opened new opportunities for medical prognostication (Assidi et al., 2022; Zhang et al., 2024; Pantanowitz et al., 2025). ML approaches can leverage large-scale clinical datasets to generate flexible, high-performance models, while the emergence of interpretable machine learning (IML) techniques allows not only prediction but also transparency in feature contribution (Ahlquist et al., 2023). Such interpretability is especially vital in high-stakes settings, such as intensive care units (Fleuren et al., 2020). The Medical Information Mart for Intensive Care IV (MIMIC-IV) database, with its extensive and well-structured data on critically ill patients, provides an ideal foundation for developing and validating ML-based risk prediction models (Johnson et al., 2023).

In this study, we harness clinical data from MIMIC-IV to construct and validate multiple ML models for predicting the risk of sepsis during hospitalization in PI patients. We further employ SHapley Additive exPlanations (SHAP) to elucidate feature importance, thereby enhancing interpretability. Beyond internal validation, we introduce an external validation cohort derived from Guangxi Medical University Cancer Hospital, comprising real-world clinical data from a tertiary care setting in China. This independent dataset enables us to rigorously evaluate model generalizability across different populations and healthcare systems. By systematically comparing model performance in terms of discrimination, calibration, and clinical net benefit, our goal is to establish a clinically applicable risk assessment tool that balances predictive accuracy with interpretability, ultimately facilitating early risk stratification and individualized management of PI patients.

## 2 Materials and methods

### 2.1 Data source

**Figure 1:**
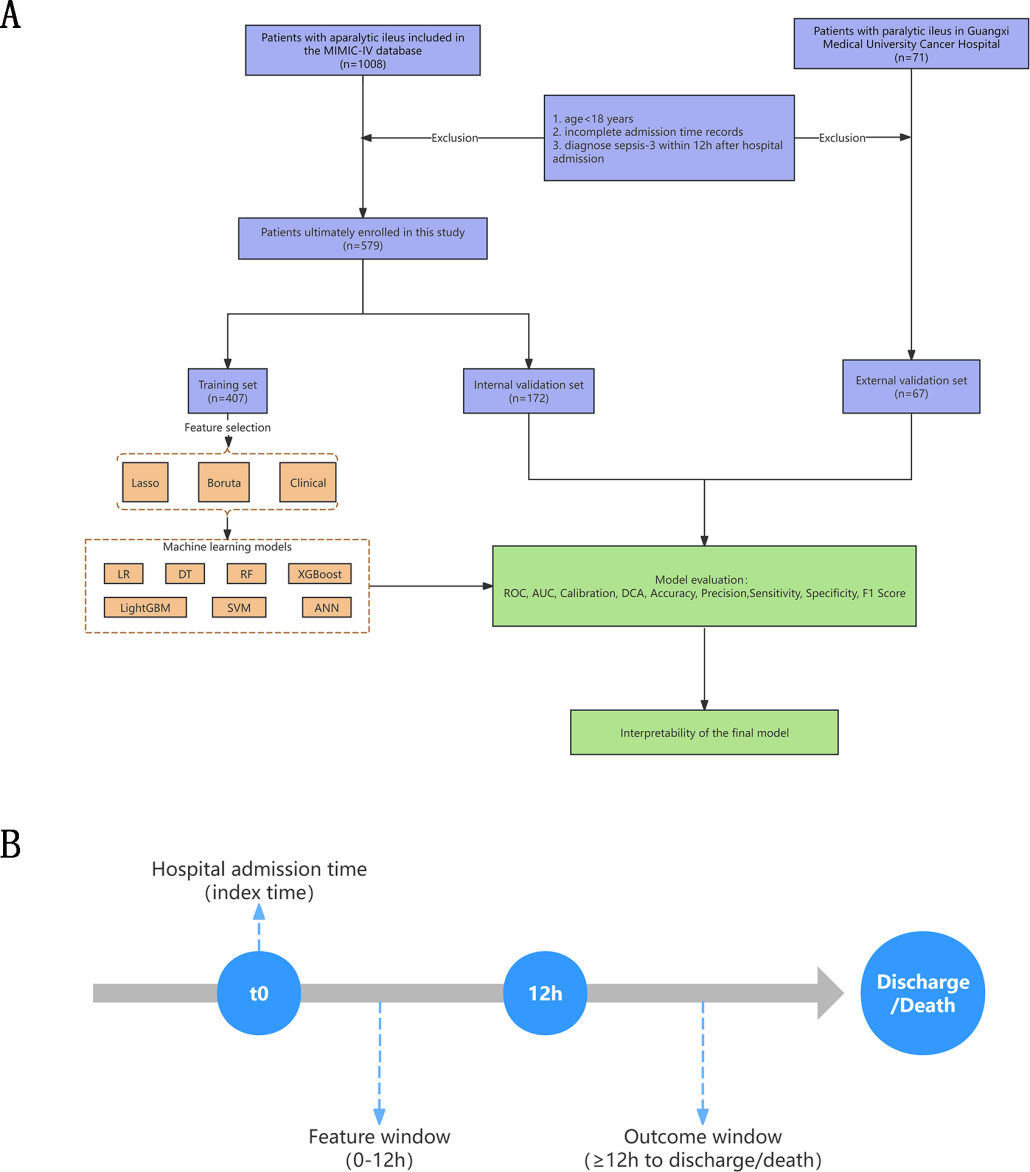
**(A)** The flowchart of patients’ selection. **(B)** Timeline of the prediction framework.

This study utilized data from the MIMIC-IV database (version 3.1), an open-access resource containing detailed clinical information on patients admitted to the Beth Israel Deaconess Medical Center (BIDMC), Boston, between 2008 and 2022 (Johnson et al., 2023). To ensure privacy, all patient identifiers, including names and timestamps, have been de-identified; therefore, informed consent was waived. Access to the database was granted after successful completion of the Collaborative Institutional Training Initiative (CITI) program by author Qingzhou Song (certification ID: 13910266). An external validation set was obtained from the Guangxi Medical University Cancer Hospital, encompassing adult patients hospitalized between January 2010 and July 2025. These data were fully de-identified prior to analysis in accordance with institutional policies and ethical approval. The external validation cohort was constructed using the same inclusion criteria, variable definitions, and feature set as those applied in the MIMIC-IV cohort, ensuring consistency between internal and external analyses. The study flowchart is shown in **Figure 1A**.

### 2.2 Study population

We included patients diagnosed with PI, identified using ICD-9 code 5601 and ICD-10 code K560. For individuals with multiple admissions, only data from the first hospitalization were analyzed. Sepsis was defined according to the Sepsis-3 criteria as life-threatening organ dysfunction caused by a dysregulated host response to infection (Singer et al., 2016). Operationally, sepsis onset was determined by: Suspected infection, defined by the temporal concurrence of systemic antibiotic administration and microbiological culture sampling; and Organ dysfunction, defined as an increase in the Sequential Organ Failure Assessment (SOFA) score of ≥2 points from baseline. The sepsis onset time was defined as the earliest time point at which both criteria were satisfied. Only sepsis events occurring within the predefined outcome window were considered valid outcomes. Patients were excluded if they: Were younger than 18 years; Had incomplete admission time records; Met Sepsis-3 diagnostic criteria within the first 12 hours after hospital admission (baseline sepsis), to avoid contamination of the prediction task.

The index time (t0) was defined as the hospital admission time. All predictor variables were extracted exclusively from a fixed feature window spanning 0–12 hours after hospital admission (t0 to t0 + 12 h). Any measurements or events occurring after t0 + 12 h were excluded from feature construction. The prediction target was new-onset sepsis occurring after the feature window. Sepsis onset was required to occur at or after t0 + 12 h (**Figure 1B**). A Cox proportional hazards model was then constructed using the same predictors derived from the 0–12 h feature window. The outcome of interest was time to first sepsis onset after the landmark time. Model performance was assessed using the concordance index (C-index) and time-dependent area under the receiver operating characteristic curve (AUC). This analysis provided an independent evaluation of early risk stratification under a time-to-event framework and served as a robustness check against residual temporal leakage.

Using structured query language (SQL), we extracted a wide range of clinical variables within the first 12 hours of hospital admission and prior literature: baseline characteristics (age, sex, height, weight), vital signs (heart rate, mean non-invasive blood pressure, respiratory rate, SpO2), laboratory parameters (hematology, liver function, renal function, coagulation, arterial blood gases), medication use (sedatives analgesics, vasopressors, antibiotics), comorbidities (atrial fibrillation, hypertension, liver cirrhosis, pneumonia, cerebrovascular accident, chronic kidney disease, cancer, diabetes, heart failure, ischemic heart disease, chronic obstructive pulmonary disease), and intervention-related variables (used only in the extended model and sensitivity analyses):mechanical ventilation, abdominal surgery. All predictors were restricted to the 0–12 h feature window. Intervention-related variables were defined as binary indicators reflecting whether the intervention occurred within this window. Given the ICU setting, missingness was considered unlikely to be completely at random, as laboratory testing is often guided by clinical condition and disease severity. Based on clinical plausibility and exploratory analyses, missing data were assumed to be predominantly missing at random (MAR), conditional on observed covariates. Variables with ≥20% missing values were excluded from further analysis. For variables with <20% missingness, missing values were imputed using multiple imputation by chained equations (MICE). Importantly, the imputation models were fitted exclusively on the training dataset using baseline outpatient and admission data. The learned imputation parameters were then applied to the internal validation and external validation datasets without re-estimation, thereby preventing information leakage across datasets.

After cohort construction and temporal filtering, the dataset was randomly split into a training set and an internal validation set at a ratio of 70:30. An independent external cohort from a tertiary medical center was used exclusively for external validation to assess model generalizability.

### 2.3 Statistical analysis

Continuous variables are presented as mean (SD) or median (IQR), as appropriate, and compared using Student’s t-test or the Wilcoxon rank-sum test, as appropriate. Categorical variables were summarized as counts and percentages, and compared using the χ² test or Fisher’s exact test. A two-tailed P ≤ 0.05 was considered statistically significant. All statistical analyses were conducted using R version 4.5.1 and Python version 3.10.4.

### 2.4 Feature selection

Feature selection was conducted exclusively in the training set using a hybrid strategy that combined data-driven algorithms with clinical domain knowledge. First, two complementary data-driven methods were applied. Least Absolute Shrinkage and Selection Operator (LASSO) regression was used to identify a parsimonious subset of predictors by applying L1 regularization, thereby reducing multicollinearity and emphasizing linear associations. In parallel, the Boruta algorithm, an all-relevant feature selection method based on random forests, was employed to capture potentially important nonlinear effects and interactions. Rather than relying on a single algorithm, we considered the union of features identified by LASSO and Boruta as the initial candidate set, in order to minimize the risk of excluding clinically meaningful predictors. This candidate set was subsequently reviewed in the context of clinical plausibility, temporal availability within the first 12 hours of admission, and potential redundancy.

### 2.5 Model development and evaluation

The primary analysis was conducted using a parsimonious set of predictors derived from demographics, comorbidities, vital signs, and laboratory variables. All predictors were restricted to data available within the first 12 hours after admission, and no intervention-related variables were included, in order to provide an early, treatment-unbiased risk assessment that reflects a clinically deployable early-risk stratification strategy. As a sensitivity analysis, a predefined group of early intervention-related variables—specifically mechanical ventilation, vasopressor use, sedative–analgesic use, antibiotic administration, and abdominal surgery—was additionally incorporated using the same 0–12 h feature window. These variables were intentionally forced into the model solely to evaluate the incremental prognostic contribution of early care pathway information, rather than to redefine the primary prediction framework.

All continuous variables were standardized to eliminate scale differences and reduce model bias. Seven machine learning algorithms were implemented: logistic regression, decision tree (DT), random forest (RF), XGBoost, LightGBM, support vector machine (SVM), and artificial neural network (ANN). Hyperparameters were optimized using grid search with fivefold cross-validation to minimize overfitting. All models were trained on the training set and evaluated on the internal and external validation sets without re-fitting. Classification thresholds were determined on the internal validation set using a predefined dual-constraint strategy. Specifically, predicted probabilities were scanned from 0.01 to 0.99, and thresholds satisfying a minimum sensitivity of 0.80 and specificity of 0.70 were considered clinically acceptable. Among these candidate thresholds, the one maximizing the F1 score was selected. This approach avoided arbitrary thresholding and reflected the clinical priority of early sepsis detection while controlling false-positive rates. Model performance was evaluated in both training and validation sets using the area under the receiver operating characteristic curve (AUC), sensitivity, specificity, accuracy, and F1 score. Calibration curves, decision curve analysis (DCA), and receiver operating characteristic (ROC) plots were also constructed. For decision curve analysis, net benefit was evaluated across a wide range of threshold probabilities (0.01–0.99). Thresholds of 10%, 20%, and 30% were highlighted as clinically plausible decision points corresponding to low-, intermediate-, and high-risk scenarios. The baseline risk used in the decision curves was derived from the observed sepsis incidence in the validation cohort. The optimal model was selected *a priori* based on the highest F1 score in the internal validation set, reflecting a balanced trade-off between sensitivity and precision in the context of early sepsis risk identification. In addition, sensitivity analyses were performed using the same set of predictors while varying the feature windows (0–24 h and 0–48 h), and by excluding patients with very early sepsis onset (within 6 h after admission), with the optimal model. Calibration and clinical net benefit were used as complementary criteria to support model robustness and clinical interpretability. For external validation, probability recalibration using intercept and slope adjustment was applied when appropriate to account for differences in baseline risk between cohorts.

### 2.6 Model interpretation

The best-performing model was further interpreted using SHAP, a game-theoretic approach that quantifies the contribution of each feature to individual predictions and the overall model. SHAP values provided both global and local interpretability, thereby enabling clinicians to better understand the rationale underlying model predictions.

## 3 Results

### 3.1 Study population

A total of 1008 patients with paralytic ileus were initially identified from the MIMIC-IV database. After excluding patients younger than 18 years, those with incomplete admission records, and those who developed sepsis within the first 12 hours, 579 patients were included in the final analysis. Among them, 226 patients (29.78%; 95% CI, 26.54–33.17%) developed new-onset sepsis after the landmark time. The cohort was randomly split into a training set (n=407) and an internal validation set (n=172). An external validation cohort consisting of 67 patients was obtained from Guangxi Medical University Cancer Hospital, of whom 26 patients (38.81%; 95% CI, 27.14–51.50%) developed sepsis.

Baseline characteristics of the study population are presented in **Table 1**. Patients in the sepsis group had a higher prevalence of atrial fibrillation and pneumonia, as well as more frequent heart failure (all P < 0.05). Several laboratory parameters differed significantly between groups, including lower platelet count, higher RDW, prolonged coagulation indices (PT and PTT), and elevated markers of renal dysfunction such as creatinine and blood urea nitrogen. No significant differences were observed in age, sex, or most electrolyte measurements. Missing data for each variable are summarized in **Supplementary Table 1**. Among patients who developed sepsis, the median time from ICU admission to sepsis onset was 91.48 hours (IQR, 44.55–174.78).

**Table 1:** Baseline characteristics of patients.

Variables	Total (n=579)	Sepsis (n=226)	Non-sepsis (n=353)	P value
Age (years)	66.00 (54.00-77.00)	67.00 (54.25-77.00)	66.00 (54.00-76.00)	0.635
Weight (kg)	82.00 (69.00-96.40)	81.68 (70.65-97.91)	82.10 (69.00-96.07)	0.405
Gender				
Female, n (%)	219 (37.82)	88 (38.94)	131 (37.11)	0.723
Male, n (%)	360 (62.18)	138 (61.06)	222 (62.89)	
Comorbidity				
AF, n (%)	169 (29.19)	79 (34.96)	90 (25.50)	0.019
Hypertension, n (%)	264 (45.60)	102 (45.13)	162 (45.89)	0.926
LC, n (%)	58 (10.02)	28 (12.39)	30 (8.50)	0.168
Pneumonia, n (%)	152 (26.25)	83 (36.73)	69 (19.55)	<0.001
CVA, n (%)	46 (7.94)	18 (7.96)	28 (7.93)	>0.999
CKD, n (%)	84 (14.51)	34 (15.04)	50 (14.16)	0.863
Cancer, n (%)	100 (17.27)	39 (17.26)	61 (17.28)	>0.999
Diabetes, n (%)	125 (21.59)	54 (23.89)	71 (20.11)	0.330
HF, n (%)	121 (20.90)	59 (26.11)	62 (17.56)	0.018
IHD, n (%)	162 (27.98)	74 (32.74)	88 (24.93)	0.051
COPD, n (%)	87 (15.03)	33 (14.60)	54 (15.30)	0.913
Interventions				
SA, n (%)	24 (4.15)	1 (0.44)	23 (6.52)	<0.001
Vasopressor, n (%)	17 (2.94)	1 (0.44)	16 (4.53)	0.010
Antibiotics, n (%)	13 (2.25)	0 (0.00)	13 (3.68)	0.009
Surgery, n (%)	234 (40.41)	84 (37.17)	150 (42.49)	0.235
Ventilation, n (%)	115 (19.86)	15 (6.64)	100 (28.33)	<0.001
Laboratory parameters				
Hematocrit (%) Ref: 36–50	31.244 (29.00-34.69)	30.82 (28.54-34.23)	31.40 (29.17-35.10)	0.067
Hemoglobin (g/dL) Ref: 12–16 (female), 13–17 (male)	10.41 (9.56-11.58)	10.38 (9.45-11.3)	10.50 (9.60-11.77)	0.060
PLT (10^9^/L) Ref: 150–400	233.25 (174.67-304.40)	225.62 (169.65-285.46)	237.00 (180.00-318.60)	0.013
RDW (%) Ref: 11.5–14.5	14.91 (14.00-16.45)	15.30 (14.23-16.92)	14.70 (13.76-16.01)	<0.001
RBC (10^12^/L) Ref: 4.0–5.5	3.51 (3.19-3.86)	3.44 (3.13-3.81)	3.55 (3.24-3.92)	0.007
WBC (10^9^/L) Ref: 4.0–10.0	9.97 (7.64-12.81)	10.11 (8.20-12.86)	9.87 (7.47-12.70)	0.910
AG (mEq/L) Ref: 8–16	13.37 (12.05-14.82)	13.53 (12.34-15.02)	13.09 (12.00-14.59)	0.364
Calcium (mg/dL) Ref: 8.6–10.2	8.42 (8.01-8.75)	8.42 (8.08-8.75)	8.40 (8.00-8.75)	0.518
Chloride (mEq/L) Ref: 98–106	103.00 (100.66-105.38)	102.25 (100.45-104.86)	103.62 (101.00-106.00)	0.079
Glucose (mg/dL) Ref: <200	123.02 (108.03-145.96)	123.25 (110.45-145.71)	122.46 (107.00-147.60)	0.408
Potassium (mEq/L) Ref: 3.5–5.0	4.11 (3.90-4.30)	4.15 (3.91-4.29)	4.10 (3.90-4.30)	0.204
Sodium (mEq/L) Ref: 135–145	138.00 (136.12-140.00)	137.83 (136.35-139.72)	138.07 (136.00-140.10)	0.550
Total CO2 (mEq/L) Ref: 22–29	25.00 (22.48-28.00)	25.88 (22.54-28.19)	24.83 (22.36-27.50)	0.286
Lactate (mmol/L) Ref: 0.5–2.0	1.73 (1.30-2.38)	1.81 (1.33-2.39)	1.67 (1.30-2.25)	0.208
PCO2 (mmHg) Ref: 35–45	40.36 (36.75-44.61)	40.36 (37.46-45.00)	40.36 (36.60-44.00)	0.332
PH Ref: 7.35–7.45	7.38 (7.34-7.42)	7.39 (7.35-7.42)	7.38 (7.34-7.42)	0.859
PO2 (mmHg) Ref: 80–100	121.95 (92.42-172.45)	113.25 (91.24-152.28)	127.00 (93.07-182.55)	0.021
INRPT Ref: 0.8–1.2	1.29 (1.13-1.66)	1.32 (1.18-1.75)	1.27 (1.18-1.53)	0.050
PT (s) Ref: 11–15	14.20 (12.96-17.99)	14.62 (13.10-18.89)	14.00 (12.88-16.92)	0.034
PTT (s) Ref: 25–35	34.59 (28.90-46.33)	37.05 (30.39-50.16)	33.30 (28.28-43.93)	0.002
ALT (IU/L) Ref: 7–40	28.00 (18.50-50.38)	29.98 (16.62-58.80)	27.57 (19.67-44.18)	0.890
AST (IU/L) Ref: 10–40	33.77 (23.28-59.00)	36.95 (24.04-77.60)	32.83 (23.00-52.67)	0.292
TBIL (mg/dL) Ref: 0.2–1.2	0.65 (0.40-1.24)	0.73 (0.44-1.59)	0.61 (0.40-1.10)	0.008
Creatinine (mg/dL) Ref: 0.6–1.3	0.99 (0.75-1.43)	1.09 (0.78-1.68)	0.92 (0.72-1.30)	0.025
BUN (mg/dL) Ref: 7–20	20.39 (14.02-31.23)	23.66 (15.19-36.30)	19.00 (13.83-28.38)	<0.001
Sodium (mEq/L) Ref: 135–145	138.00 (136.12-140.00)	137.83 (136.35-139.72)	138.07 (136.00-140.10)	0.550
Free Calcium (mmol/L) Ref: 1.12–1.32	1.11 (1.06-1.15)	1.11 (1.06-1.15)	1.11 (1.06-1.16)	0.217

AF, Atrial Fibrillation; LC, Liver Cirrhosis; CVA, Cerebrovascular Accident; CKD, Chronic Kidney Disease; HF, Heart Failure; IHD, Ischemic Heart Disease; COPD, Chronic Obstructive Pulmonary Disease; SA, Sedative Analgesics; PLT, Platelet; RDW, Red Cell Distribution Width; RBC, Red Blood Cell; WBC, White Blood Cell; AG, Anion Gap; INRPT, International Normalized Ratio of Prothrombin Time; PT, Prothrombin Time; PTT, Partial Thromboplastin Time; ALT, Alanine Aminotransferase; AST, Aspartate Aminotransferase; TBIL, Total Bilirubin; BUN, Blood Urea Nitrogen.

Antibiotic use was defined as administration within the first 24 hours after admission. Patients who developed sepsis within the first 24 hours were excluded by study design. Therefore, no antibiotic exposure was observed during the prediction window among patients who later developed sepsis, reflecting early pre-diagnostic clinical management rather than data extraction error. Vital signs measured within the first 12 hours after admission were not included because more than 20% of these variables had missing values and were therefore excluded according to the predefined missing-data threshold.

### 3.2 Feature selection

**Figure 2:**
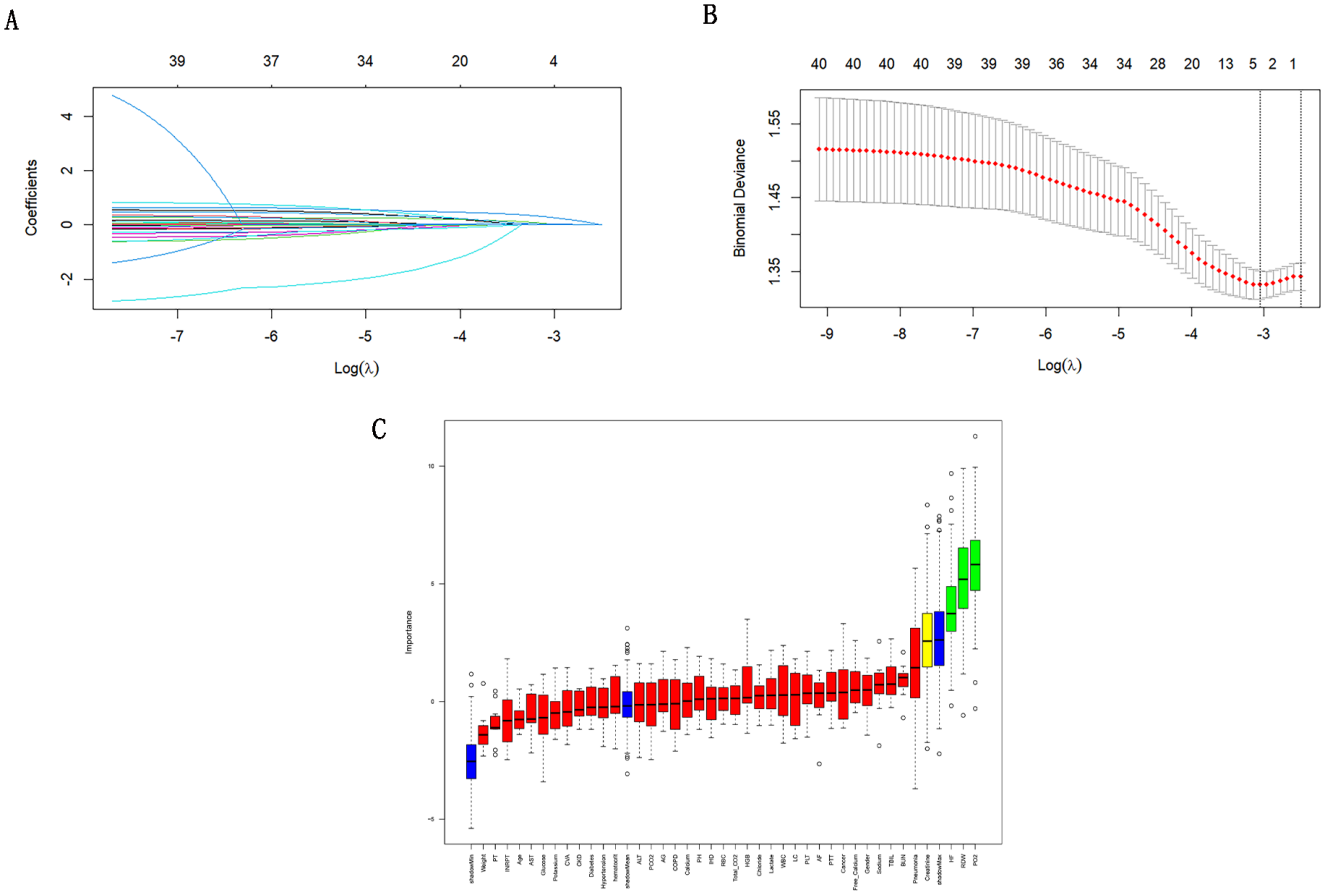
Feature selection results: **(A)** coefficient profiles of candidate variables plotted against the log-transformed regularization parameter (log λ). **(B)** Ten-fold cross-validation curve showing binomial deviance versus log λ. The vertical dashed lines indicate the optimal values of λ (λ_min and λ_1se). Numbers above the plot represent the number of nonzero coefficients. **(C)** Variables selected using the Boruta algorithm, identifying relevant predictors for sepsis in paralytic ileus.

Two complementary feature selection approaches—LASSO regression and the Boruta algorithm—were applied to identify predictors of sepsis. In LASSO regression, the optimal regularization parameter (λ) was determined through tenfold cross-validation. At λmin = 0.04707123, 4 variables were retained (coefficients shown in **Supplementary Table 2**; **Figures 2A, B**). The Boruta algorithm similarly identified 3 relevant predictors (**Supplementary Table 3**; **Figure 2C**). The intersection of these methods resulted in 2 key predictors: heart failure and RDW. Additionally, based on clinical prior knowledge and existing evidence-based medical research, pneumonia, blood urea nitrogen (BUN), atrial fibrillation (AF), chloride (Cl), and white blood cell (WBC) count have been consistently identified as independent predictors of both the development and prognosis of sepsis. Pneumonia represents one of the most common sources of infection leading to sepsis (Mayr et al., 2014). Elevated BUN levels typically indicate impaired renal function or a state of systemic hypoperfusion (Dhondup and Qian, 2017). The occurrence of atrial fibrillation reflects an abnormal cardiovascular response to infection-related stress (Leng et al., 2023). Alterations in serum chloride concentration are closely associated with acid–base imbalance and electrolyte disturbances (Popović et al., 2025), whereas an increased WBC count remains one of the most classical and sensitive markers of infection-induced inflammatory response (Ho et al., 2023). Collectively, incorporating these five variables into the predictive model enables comprehensive characterization of patients across three critical dimensions—infection source, physiological status, and immune response—thereby facilitating a more accurate and holistic assessment of the risk of progression to sepsis in patients with paralytic ileus.

### 3.3 Model development and evaluation

**Figure 3:**
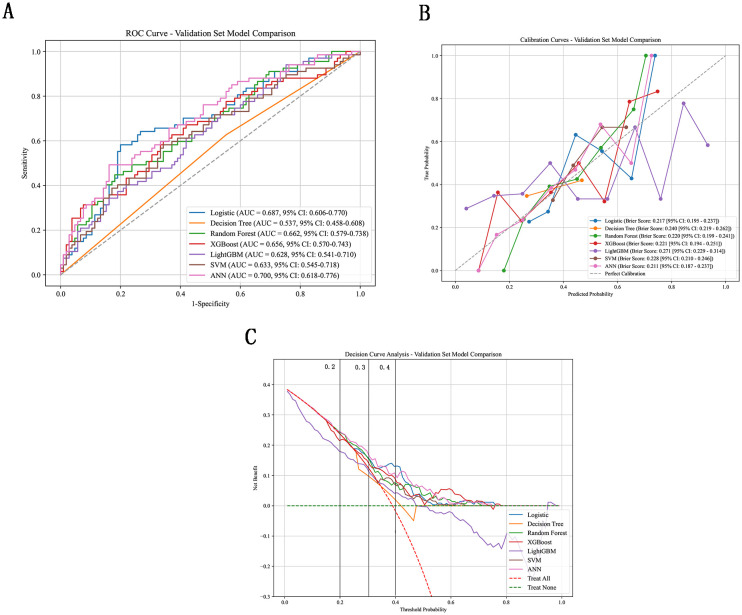
Performance of machine learning models in the internal validation set: **(A)** receiver operating characteristic (ROC) curve showing the discriminative ability of the machine learning models. **(B)** Calibration curve comparing predicted probabilities to actual outcomes. **(C)** Decision curve analysis (DCA) curve illustrating the net clinical benefit of the machine learning models across different threshold probabilities.

The predictive performance of the seven machine learning models in the internal validation cohort is summarized in **Table 2**. Overall, model discrimination ranged from modest to acceptable, with AUC values between 0.537 and 0.700. Logistic regression demonstrated stable performance and was selected as the final model due to its balanced classification ability and clinical interpretability. Specifically, logistic regression achieved an AUC of 0.687 (95% CI: 0.606–0.770) (**Figure 3A**), with an accuracy of 0.709 and an F1 score of 0.609. At the selected threshold, the model yielded a sensitivity of 0.582 and a specificity of 0.790, indicating an acceptable trade-off between early sepsis detection and false-positive control.

**Table 2:** Performance evaluation of seven algorithmic models on the internal validation set.

Model	AUC	95% CI lower	95% CI upper	Accuracy	Precision	Sensitivity	Specificity	F1 score
Logistic	0.687	0.606	0.770	0.709	0.639	0.582	0.790	0.609
Decision Tree	0.537	0.458	0.608	0.517	0.420	0.627	0.448	0.503
Random Forest	0.662	0.579	0.738	0.669	0.600	0.448	0.810	0.513
XGBoost	0.656	0.570	0.743	0.628	0.519	0.597	0.648	0.556
LightGBM	0.628	0.541	0.710	0.593	0.482	0.612	0.581	0.539
SVM	0.633	0.545	0.718	0.628	0.520	0.582	0.657	0.549
ANN	0.700	0.618	0.776	0.698	0.647	0.493	0.829	0.559

Calibration analysis (**Figure 3B**) revealed that ANN, logistic regression, and RF had predicted probabilities that closely approximated the ideal diagonal, indicating good calibration. The DT and LightGBM models demonstrated more noticeable deviations, suggesting they were less reliable in estimating true probabilities.

DCA (**Figure 3C**) further illustrated that ANN, logistic regression, and RF provided superior net clinical benefit across a wide range of threshold probabilities, compared with the “treat all” and “treat none” strategies. XGBoost also provided a favorable net benefit, while DT consistently underperformed at higher thresholds.

**Figure 4:**
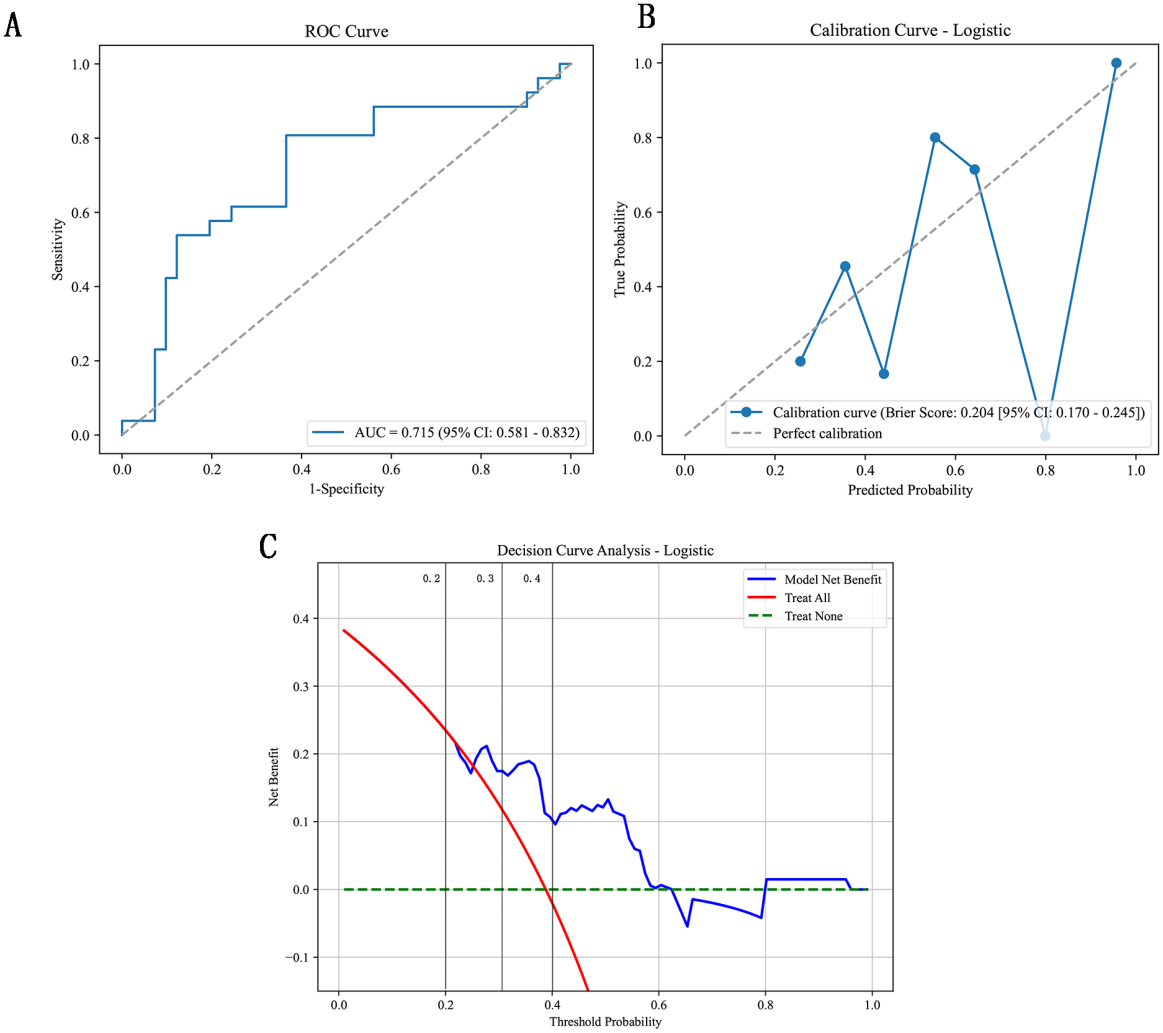
External validation of the final logistic regression model: **(A)** ROC curve. **(B)** Calibration curve. **(C)** DCA curve.

The generalizability of the final logistic regression model was further assessed in an independent external validation cohort (**Figures 4A–C**). The model maintained acceptable discriminatory ability, achieving an AUC of 0.715 (95% CI: 0.581–0.832), which was comparable to its performance in the internal validation set. At the predefined classification threshold, logistic regression yielded an accuracy of 0.701 and demonstrated high sensitivity (0.808), indicating a strong capability to identify patients at risk of developing sepsis in a different clinical setting. Calibration analysis in the external cohort showed good consistency between predicted and observed sepsis probabilities, suggesting that the model preserved reliable risk estimation across populations (**Figure 4B**). In addition, decision curve analysis confirmed that logistic regression provided a positive net benefit over treat-all and treat-none strategies across a range of clinically relevant threshold probabilities (**Figure 4C**).

Sensitivity analyses were conducted to evaluate the robustness of the primary logistic regression model under alternative feature windows and modeling specifications (**Supplementary Table 4**). When extending the feature extraction window from 0–12 h to 0–24 h and 0–48 h after admission, the model maintained acceptable discriminatory ability, with internal validation AUCs of 0.651 and 0.665, respectively. Similarly, applying a stricter exclusion criterion by removing patients who developed sepsis within the first 6 hours resulted in an AUC of 0.602, indicating that the predictive performance remained relatively stable despite changes in outcome definition. In addition, an extended model (Sens-4) incorporating early intervention–related variables, including mechanical ventilation, vasopressor use, sedative–analgesic use, antibiotic administration, and abdominal surgery, achieved an improved AUC of 0.752, with comparable calibration performance. Decision curve summaries across sensitivity settings consistently demonstrated positive net benefit over clinically relevant threshold probability ranges. As a complementary analysis, a Cox proportional hazards model yielded a C-index of 0.588 and a 24-hour time-dependent AUC of 0.582, further supporting the feasibility of early sepsis risk stratification using admission-level data.

Additional discrimination, calibration, and decision metrics of the final logistic regression model are summarized in **Supplementary Table 5**. In the external validation cohort, the model demonstrated comparable discrimination with an AUC of 0.715 and a Brier score of 0.204 before recalibration. The calibration intercept remained close to zero (0.025), while the slope was 1.208, indicating modest differences in risk distribution between cohorts. After probability recalibration, overall discrimination was preserved (AUC 0.715), and the Brier score slightly improved to 0.203. Sensitivity decreased from 0.808 to 0.654, whereas specificity remained stable (0.634), reflecting a more conservative risk estimation after adjustment.

The clinical implications of different risk thresholds in the external validation cohort are shown in **Supplementary Table 6**. Lower thresholds (0.1–0.2) maximized sensitivity but produced very low specificity, resulting in more false-positive classifications. At intermediate thresholds (e.g., 0.3–0.4), the model achieved a more balanced trade-off between sensitivity and specificity. Higher thresholds further increased specificity and positive predictive value, with a reduced number needed to treat, highlighting the impact of threshold selection on clinical decision-making.

Baseline characteristics of the internal (MIMIC-IV) and external validation cohorts are summarized in **Supplementary Table 7**. The external cohort was younger and had a higher proportion of sedative–analgesic use compared with the MIMIC-IV cohort (P < 0.05). No significant differences were observed in sex distribution, vasopressor use, antibiotic use, abdominal surgery, or mechanical ventilation (all P > 0.05).

Additional benchmarking against conventional clinical scores was performed in the internal validation cohort. As shown in **Supplementary Table 8** and **Supplementary Figure 1**, the proposed logistic regression model achieved an AUC of 0.687, which was higher than SIRS, SAPS II, and APS III, with AUCs of 0.616, 0.669, and 0.668, respectively. SIRS showed high sensitivity but low specificity, whereas SAPS II showed high specificity but limited sensitivity. The logistic regression model achieved the highest F1 score and a comparable Brier score, with positive net benefit across a threshold probability range of approximately 0.25–0.75.

### 3.4 Model interpretability (SHAP analysis)

**Figure 5:**
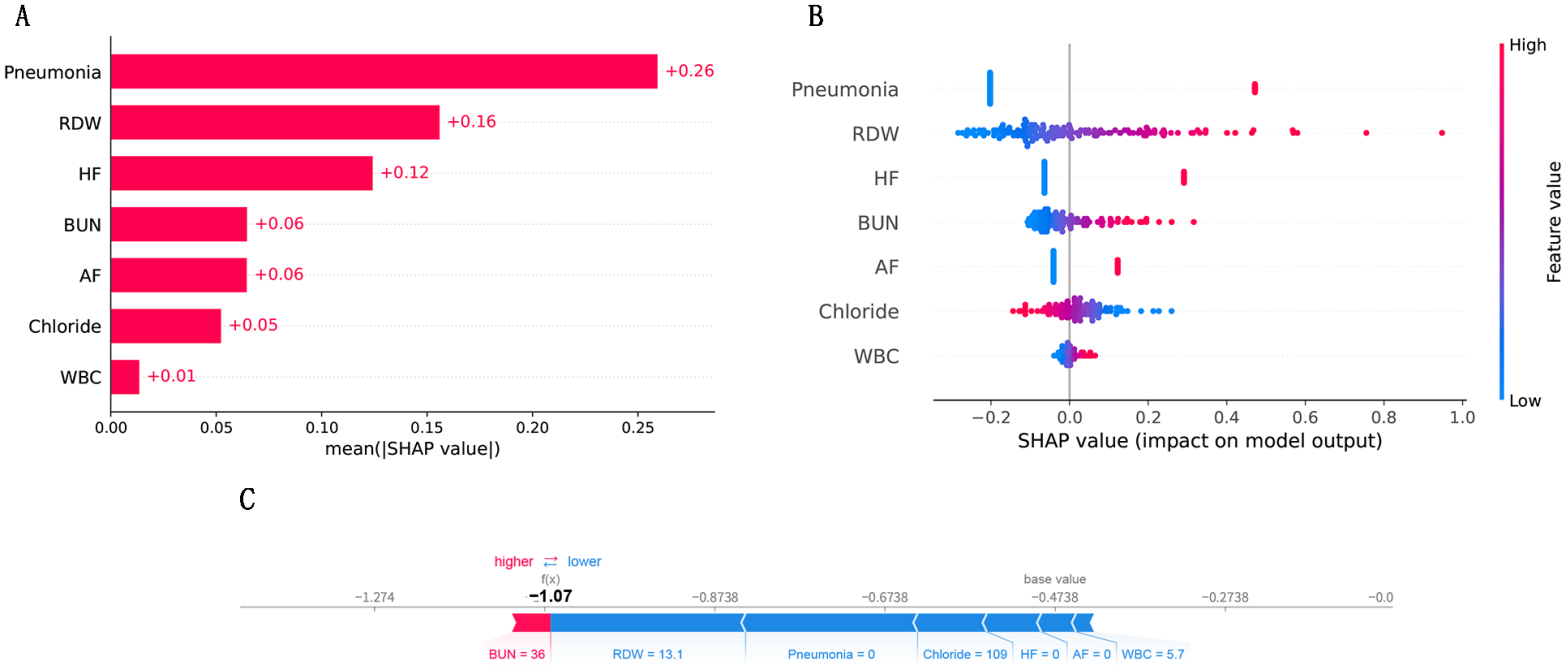
SHAP analysis results: **(A)** SHAP Importance Plot showing the average absolute SHAP values for each feature, highlighting the most influential predictors in the model. **(B)** SHAP beeswarm depicting the distribution of SHAP values for each feature, illustrating the relationship between feature values and model predictions for sepsis risk. **(C)** SHAP force plot for a representative patient, demonstrating how individual features contribute to the final model prediction relative to the baseline value.

To enhance the interpretability of the optimal logistic regression model, SHAP were applied to quantify the contribution of each feature to the prediction of sepsis in patients with PI. The SHAP summary bar plot demonstrated that pneumonia was the most influential predictor, with the highest mean absolute SHAP value (0.26), followed by red cell distribution width (RDW, 0.16), heart failure (HF, 0.12), blood urea nitrogen (BUN, 0.06), atrial fibrillation (AF, 0.06), serum chloride (0.05), and white blood cell count (WBC, 0.01) (**Figure 5A**).

The SHAP beeswarm plot further illustrated the direction and magnitude of feature effects (**Figure 5B**). Higher values of RDW and BUN were predominantly associated with positive SHAP values, indicating an increased predicted risk of sepsis. The presence of pneumonia, HF, and AF also contributed positively to the model output. In contrast, higher chloride levels tended to exhibit negative SHAP values, suggesting a protective association with sepsis risk. The impact of WBC on the model prediction was relatively limited.

An individual-level SHAP force plot was used to explain the model’s prediction for a representative patient (**Figure 5C**). In this case, elevated BUN and RDW were the primary contributors increasing the predicted probability of sepsis, whereas the absence of pneumonia, HF, and AF, along with higher chloride levels, reduced the predicted risk. These findings indicate that both comorbidities and laboratory indicators jointly influenced the model’s prediction.

**Figure 6:**
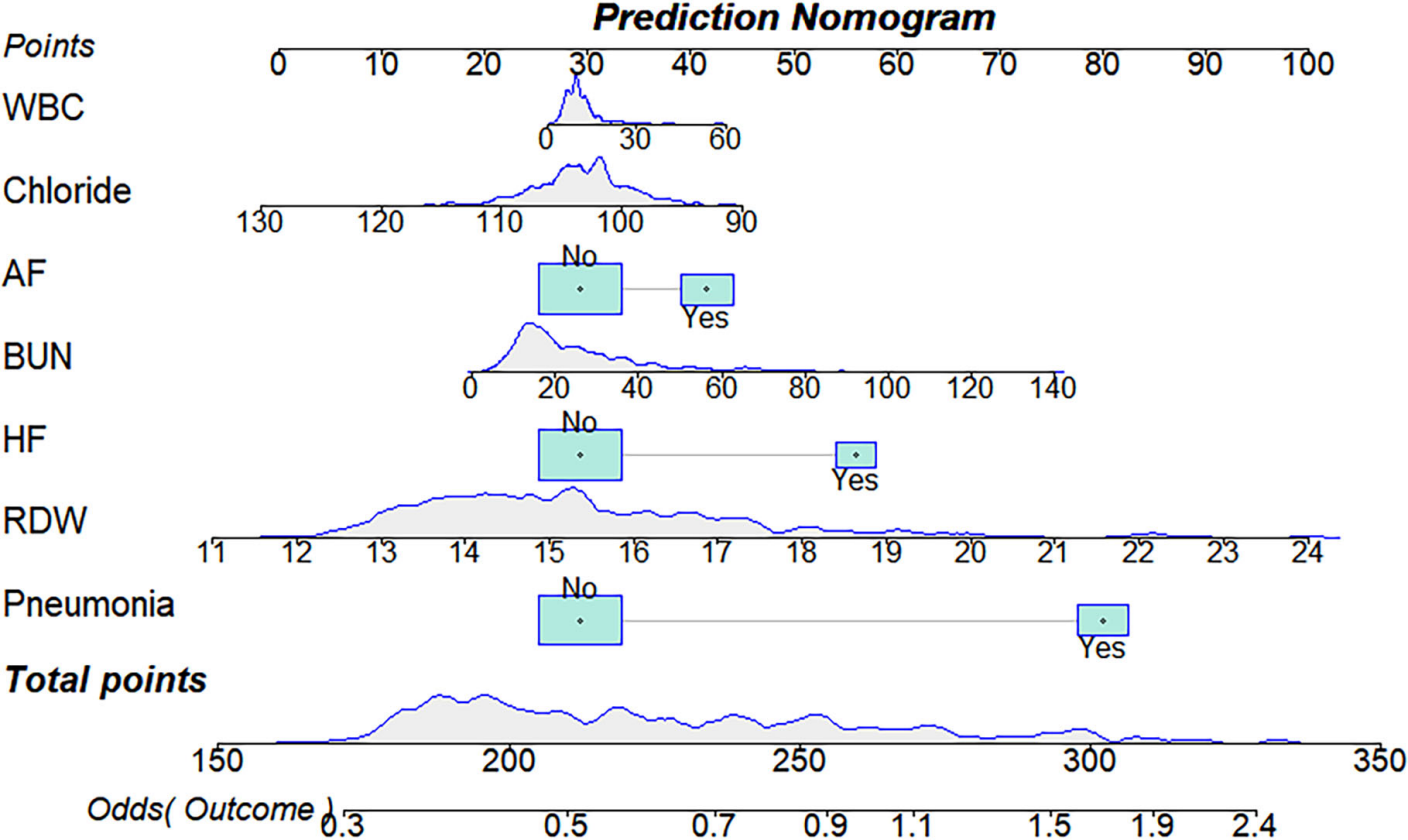
Nomogram for individualized prediction of sepsis.

Based on the optimal logistic regression model, a nomogram was constructed to provide an individualized prediction of sepsis in patients with PI (**Figure 6**). Each predictor was assigned a weighted score according to its regression coefficient, and the total points were obtained by summing the individual scores across all variables. The total score corresponded to the predicted odds of developing sepsis. Among the included predictors, pneumonia, RDW, and HF contributed the largest point allocations, whereas WBC showed a relatively smaller contribution.

## 4 Discussion

In this study, we developed and externally validated an early prediction model for new-onset sepsis in patients with PI using routinely available clinical data within the first 12 hours of hospital admission. By integrating machine learning–based feature selection with clinically informed modeling, we identified a parsimonious set of seven predictors and demonstrated that a logistic regression model achieved stable discrimination, good calibration, and consistent net clinical benefit across internal and external cohorts. These findings suggest that early risk stratification for sepsis in PI patients is feasible before overt clinical deterioration or therapeutic escalation occurs.

PI is frequently accompanied by systemic inflammation, bacterial translocation, and impaired host defense, all of which increase susceptibility to sepsis (Stein et al., 2018; Ma et al., 2023). Existing sepsis prediction models may not necessarily transport well to PI patients because of differences in case-mix, baseline risk, predictor distributions, intervention pathways, and temporal availability of predictors. Therefore, empirical benchmarking in the PI population is required rather than assuming poor applicability. In contrast, our study specifically focuses on patients with PI and restricts predictors to the first 12 hours after admission, a clinically meaningful window during which proactive monitoring and intervention may still modify outcomes. To our knowledge, few studies have constructed and externally validated an early sepsis prediction model tailored to this specific population. Although several machine learning algorithms were evaluated, logistic regression demonstrated comparable or superior overall performance relative to more complex models. This finding is consistent with prior evidence showing that, in moderately sized clinical datasets, simpler models often generalize better and are less prone to overfitting (Babyak, 2004; Semenova et al., 2023). Importantly, logistic regression offers superior transparency and interpretability, which are critical for clinical acceptance and implementation and align well with the priorities of risk stratification rather than black-box prediction.

The predictors included in the final model—pneumonia, RDW, HF, BUN, AF, serum chloride, and WBC—capture complementary dimensions of infection source, physiological reserve, organ dysfunction, and inflammatory response. Pneumonia emerged as the strongest contributor to sepsis risk, consistent with its role as one of the most common infectious triggers of sepsis and its frequent coexistence with gastrointestinal dysmotility (Montull et al., 2016). RDW was among the most influential laboratory predictors. Elevated RDW reflects disordered erythropoiesis, systemic inflammation, oxidative stress, and impaired physiological reserve, all of which have been linked to adverse outcomes in critically ill and septic patients (Aslam et al., 2023). Its strong association with sepsis risk in our model supports growing evidence that RDW is not merely a hematologic parameter but an integrated marker of systemic vulnerability (Zhang L. et al., 2020; Krishna et al., 2021). Cardiovascular comorbidities, including HF and AF, likely reflect limited hemodynamic adaptability to infectious stress, predisposing patients to tissue hypoperfusion and organ dysfunction (Desai et al., 2019; Liu et al., 2024; Zhu et al., 2025). Elevated BUN may indicate renal impairment, hypovolemia, or systemic hypoperfusion, all of which are early features of sepsis pathophysiology (Han et al., 2022). Interestingly, higher serum chloride levels were associated with a lower predicted risk of sepsis, possibly reflecting a more stable acid–base and electrolyte status, whereas hypochloremia may be a marker of disease severity or gastrointestinal losses (Do et al., 2022). Although WBC is a classical marker of infection, its relatively modest contribution highlights the limitations of relying on single inflammatory markers and underscores the value of a multivariable approach (Saxena et al., 2024).

From a clinical standpoint, the proposed model offers several practical advantages. First, it relies exclusively on routinely collected variables that are readily available early after admission, enabling seamless integration into clinical workflows without additional testing or cost. Second, decision curve analysis demonstrated consistent net benefit across a wide range of clinically relevant threshold probabilities, suggesting that the model could support individualized risk-based strategies, such as intensified surveillance, early diagnostic evaluation, or targeted preventive measures in high-risk patients. Third, the construction of a nomogram further enhances usability by providing an intuitive tool for bedside risk estimation and shared clinical decision-making.

Importantly, the primary model intentionally excluded intervention-related variables to preserve a treatment-unbiased prediction framework. Sensitivity analyses showed that incorporating early intervention variables improved discrimination but did not materially alter calibration, supporting the robustness of the core predictors and confirming that early baseline risk can be meaningfully assessed before therapeutic escalation.

This study has several strengths, including a landmark-based design to minimize temporal leakage, rigorous feature selection combining machine learning and clinical reasoning, comprehensive evaluation using discrimination, calibration, and decision curve analysis, and independent external validation across different healthcare settings. Nevertheless, several limitations should be acknowledged. First, the retrospective design may introduce residual confounding despite careful cohort construction. Second, the external validation cohort was relatively small, which may limit the precision of performance estimates. Third, although missing data were handled using multiple imputation under a missing-at-random assumption, unmeasured biases cannot be fully excluded. Forth, dynamic changes in clinical variables beyond the initial 12-hour window were not considered and may provide additional predictive value. Fifth, differences in patient characteristics and institutional care pathways, such as sedation strategies and infection management practices, may exist between centers and could influence the distribution of certain treatment-related variables. These factors were not explicitly modeled in the present study. Nevertheless, treatment-related variables were included as clinically meaningful indicators of illness severity rather than as causal interventions, and the model demonstrated consistent performance in the external validation cohort. In addition, granular information on antibiotic use—such as distinguishing empirical from therapeutic administration and confirming whether cultures were obtained before antibiotics—is not consistently available in the MIMIC-IV database. Therefore, antibiotic-related variables may be subject to misclassification and were not included in the primary model (only explored in sensitivity analyses) to preserve a treatment-unbiased early risk assessment.

Another limitation of this study is that we did not perform exact head-to-head implementation of all previously published machine-learning sepsis prediction models. However, many of these models require dense longitudinal EHR data, high-resolution bedside monitoring signals, unavailable engineered predictors, or target outcomes different from new-onset sepsis. Implementing substantially modified versions of these models would not constitute a valid external validation of the original models. We therefore selected fully reproducible traditional scores as clinically interpretable benchmarks.

Future research should focus on prospective, multicenter validation to further assess generalizability and clinical impact. Incorporating dynamic or time-updated variables may enhance predictive performance, and evaluating whether model-guided interventions can reduce sepsis incidence or improve outcomes will be essential. Integration of this model into electronic health record systems may facilitate automated early warning and support timely, personalized preventive strategies for patients with PI.

## 5 Conclusion

This study developed and externally validated an early prediction model for new-onset sepsis in patients with paralytic ileus using routinely available clinical data within the first 12 hours of admission. A logistic regression model based on seven key predictors demonstrated stable discrimination, good calibration, and consistent clinical net benefit across both internal and external cohorts. The proposed model provides an interpretable and practical tool for early risk stratification, which may support timely monitoring and preventive strategies in high-risk patients. Further prospective multicenter studies are warranted to confirm its clinical utility and impact on outcomes.

## Data Availability

Publicly available datasets were analyzed in this study. This data can be found here: Medical Information Mart for Intensive Care IV (MIMIC-IV) (https://physionet.org/).
