# Supplementary material for: Machine learning prediction of sepsis in paralytic ileus using interpretable clinical models
Source: Front Cell Infect Microbiol. 2026 Jun 2;16:1705126. doi: 10.3389/fcimb.2026.1705126 (PMC13269040; doi:10.3389/fcimb.2026.1705126)
Supplement: Supplementary file 1 [file DataSheet1.docx]

| Variables | Missing Values ( %) |
| --- | --- |
| Hematocrit | 0.0 |
| Hemoglobin | 0.0 |
| PLT | 0.0 |
| RDW | 0.0 |
| RBC | 0.0 |
| WBC | 0.0 |
| AG | 0.0 |
| Calcium | 0.0 |
| Chloride | 0.0 |
| Globulin | 68.0 |
| Glucose | 0.0 |
| Potassium | 0.0 |
| Sodium | 0.0 |
| Total CO2 | 8.8 |
| Free Calcium | 15.9 |
| Lactate | 2.8 |
| PCO2 | 8.8 |
| PH | 5.5 |
| PO2 | 8.8 |
| INRPT | 0.3 |
| PT | 0.3 |
| PTT | 0.3 |
| ALT | 5.5 |
| AST | 5.5 |
| TBIL | 6.7 |
| Creatinine | 0.0 |
| BUN | 0.0 |
| HR | 69.6 |
| Mean NIBP | 73.4 |
| RR | 69.6 |
| SpO2 | 69.8 |
| Temperature | 71.2 |
| Age | 0.0 |
| Weight | 3.6 |
| Height | 51.6 |

AF: Atrial Fibrillation; LC: Liver Cirrhosis; CVA: Cerebrovascular Accident; CKD: Chronic Kidney Disease; HF: Heart Failure; IHD: Ischemic Heart Disease; COPD: Chronic Obstructive Pulmonary Disease; SA: Sedative Analgesics; PLT: Platelet; RDW: Red Cell Distribution Width; RBC: Red Blood Cell; WBC: White Blood Cell; AG: Anion Gap; INRPT: International Normalized Ratio of Prothrombin Time; PT: Prothrombin Time; PTT: Partial Thromboplastin Time; ALT: Alanine Aminotransferase; AST: Aspartate Aminotransferase; TBIL: Total Bilirubin; BUN: Blood Urea Nitrogen; HR: Heart Rate; Mean NIBP: Mean Non-Invasive Blood Pressure RR: Respiratory Rate;.

Supplementary Table 1. The proportion of missing values for variables in this study

| Variables | Coefficient values |
| --- | --- |
| AF | 0 |
| Hypertension | 0 |
| LC | 0 |
| Pneumonia | 0.289202913730545 |
| CVA | 0 |
| CKD | 0 |
| Cancer | 0 |
| Diabetes | 0 |
| HF | 0.0417241333745358 |
| IHD | 0 |
| COPD | 0 |
| Gender | 0 |
| Hematocrit | 0 |
| HGB | 0 |
| PLT | 0 |
| RDW | 0.0474707411529571 |
| RBC | 0 |
| WBC | 0 |
| AG | 0 |
| Chloride | 0 |
| Glucose | 0 |
| Potassium | 0 |
| Sodium | 0 |
| Creatinine | 0 |
| BUN | 0.000603477283988196 |
| Age | 0 |
| Weight | 0 |
| Calcium | 0 |
| Free Calcium | 0 |
| Total CO2 | 0 |
| Lactate | 0 |
| PCO2 | 0 |
| PO2 | 0 |
| INRPT | 0 |
| PT | 0 |
| PTT | 0 |
| ALT | 0 |
| AST | 0 |
| TBIL | 0 |
| PH | 0 |

AF: Atrial Fibrillation; LC: Liver Cirrhosis; CVA: Cerebrovascular Accident; CKD: Chronic Kidney Disease; HF: Heart Failure; IHD: Ischemic Heart Disease; COPD: Chronic Obstructive Pulmonary Disease; SA: Sedative Analgesics; PLT: Platelet; RDW: Red Cell Distribution Width; RBC: Red Blood Cell; WBC: White Blood Cell; AG: Anion Gap; INRPT: International Normalized Ratio of Prothrombin Time; PT: Prothrombin Time; PTT: Partial Thromboplastin Time; ALT: Alanine Aminotransferase; AST: Aspartate Aminotransferase; TBIL: Total Bilirubin; BUN: Blood Urea Nitrogen

Supplementary Table 2. Coefficient values of all variables in the Lasso regression analysis

| Variables | Decision |
| --- | --- |
| HF | Confirmed |
| RDW | Confirmed |
| PO2 | Confirmed |
| AF | Rejected |
| Hypertension | Rejected |
| LC | Rejected |
| Pneumonia | Rejected |
| CVA | Rejected |
| CKD | Rejected |
| Cancer | Rejected |
| Diabetes | Rejected |
| IHD | Rejected |
| COPD | Rejected |
| Gender | Rejected |
| Hematocrit | Rejected |
| HGB | Rejected |
| PLT | Rejected |
| RBC | Rejected |
| WBC | Rejected |
| AG | Rejected |
| Chloride | Rejected |
| Glucose | Rejected |
| Potassium | Rejected |
| Sodium | Rejected |
| Creatinine | Rejected |
| BUN | Rejected |
| Age | Rejected |
| Weight | Rejected |
| Calcium | Rejected |
| Free Calcium | Rejected |
| Total CO2 | Rejected |
| Lactate | Rejected |
| PCO2 | Rejected |
| INRPT | Rejected |
| PT | Rejected |
| PTT | Rejected |
| ALT | Rejected |
| AST | Rejected |
| TBIL | Rejected |
| PH | Rejected |
| RR | Rejected |
| COPD | Rejected |
| Cancer | Rejected |
| CVA | Rejected |
| Glucose | Rejected |
| IHD | Rejected |
| T | Rejected |
| Diabetes | Rejected |
| Potassium | Rejected |

AF: Atrial Fibrillation; LC: Liver Cirrhosis; CVA: Cerebrovascular Accident; CKD: Chronic Kidney Disease; HF: Heart Failure; IHD: Ischemic Heart Disease; COPD: Chronic Obstructive Pulmonary Disease; SA: Sedative Analgesics; PLT: Platelet; RDW: Red Cell Distribution Width; RBC: Red Blood Cell; WBC: White Blood Cell; AG: Anion Gap; INRPT: International Normalized Ratio of Prothrombin Time; PT: Prothrombin Time; PTT: Partial Thromboplastin Time; ALT: Alanine Aminotransferase; AST: Aspartate Aminotransferase; TBIL: Total Bilirubin; BUN: Blood Urea Nitrogen

Supplementary Table 3. Variables selected by the BORUTA algorithm.

| Analysis set | Feature window | Early sepsis excluded | Model | AUC (internal) | Brier (internal) | DCA summary (internal) |
| --- | --- | --- | --- | --- | --- | --- |
| Primary | 0–12h | <12h | Logistic | 0.687 (0.606,0.770) | 0.217 (0.197,0.238) | 0.25-0.75 |
| Sens-1 | 0–24h | <24h | Logistic | 0.651 (0.563,0.734) | 0.219 (0.195,0.248) | 0.15-0.50 |
| Sens-2 | 0–48h | <48h | Logistic | 0.665 (0.574,0.754) | 0.205 (0.172,0.237) | 0.10-0.45 |
| Sens-3 | 0–12h | <6h | Logistic | 0.602 (0.513,0.696) | 0.190 (0.160,0.219) | 0.15-0.45 |
| Sens-4 | 0–12h | <12h | Logistic | 0.752 (0.676 - 0.817) | 0.195 (0.174 - 0.217) | 0.05-0.60 |

Sens-4: extended the primary model by forcibly incorporating early intervention–related variables, including mechanical ventilation, vasopressor use, sedative–analgesic use, antibiotic administration, and abdominal surgery.

Supplementary Table 4. Sensitivity analyses of model performance under alternative feature windows and modeling specifications.

| Cohort | AUC | Brier score | Calibration intercept | Calibration slope | E/O ratio | Optimal threshold | Sensitivity | Specificity | F1 score |
| --- | --- | --- | --- | --- | --- | --- | --- | --- | --- |
| Internal validation | 0.687 (0.606,0.770) | 0.217 (0.197,0.238) | 0.167 | 1.360 | 0.996 | 0.410 | 0.582 | 0.790 | 0.609 |
| External validation (before recalibration) | 0.715 (0.581,0.832) | 0.204 (0.170,0.245) | 0.025 | 1.208 | 1.055 | 0.360 | 0.808 | 0.634 | 0.677 |
| External validation (after recalibration) | 0.715 (0.579,0.846) | 0.203 (0.161,0.247) | 0.025 | 1.208 | 1.000 | 0.360 | 0.654 | 0.634 | 0.586 |

Supplementary Table 5. Performance, calibration, and decision metrics of the final model in the validation cohorts.

| Risk threshold | TP | FP | FN | TN | Sensitivity | Specificity | PPV | NPV | NNT† |
| --- | --- | --- | --- | --- | --- | --- | --- | --- | --- |
| 0.1 | 26 | 41 | 0 | 0 | 1.000 | 0.000 | 0.388 | 0.000 | 2.577 |
| 0.2 | 26 | 41 | 0 | 0 | 1.000 | 0.000 | 0.388 | 0.000 | 2.577 |
| 0.3 | 21 | 21 | 5 | 20 | 0.808 | 0.488 | 0.500 | 0.800 | 2.000 |
| 0.4 | 16 | 15 | 10 | 26 | 0.615 | 0.634 | 0.516 | 0.722 | 1.937 |
| 0.5 | 14 | 5 | 12 | 36 | 0.538 | 0.878 | 0.737 | 0.750 | 1.357 |

† NNT was calculated as (TP + FP) / TP.

Supplementary Table 6. Clinical consequences at predefined risk thresholds in the external validation cohort

| Variables | External Validation(n=67) | MIMIC-IV(n=579) | P value |
| --- | --- | --- | --- |
| Age (years) | 53.00 (45.00-64.00) | 66.00 (54.00-77.00) | <0.001 |
| Gender | | | |
| Female, n (%) | 29 (43.28) | 219 (37.82) | 0.461 |
| Male, n (%) | 38 (56.72) | 360 (62.18) |  |
| SA, n (%) | 9 (13.43) | 24 (4.15) | 0.003 |
| Vasopressor, n (%) | 2 (2.99) | 17 (2.94) | >0.999 |
| Antibiotics, n (%) | 2 (2.99) | 13 (2.25) | >0.999 |
| Surgery, n (%) | 25 (37.31) | 234 (40.41) | 0.720 |
| Ventilation, n (%) | 10 (14.93) | 115 (19.86) | 0.421 |

Supplementary Table 7. Baseline characteristics of patients in the internal (MIMIC-IV) cohort and the external validation cohort.

| Model | AUC | Sensitivity | Specificity | PPV | NPV | F1 | Brier score | DCA summary |
| --- | --- | --- | --- | --- | --- | --- | --- | --- |
| Logistic | 0.687 | 0.582 | 0.790 | 0.650 | 0.750 | 0.609 | 0.217 | 0.25-0.75 |
| SIRS | 0.616 | 0.791 | 0.429 | 0.469 | 0.763 | 0.589 | 0.228 | 0.20-0.45 |
| SAPSII | 0.669 | 0.373 | 0.876 | 0.658 | 0.687 | 0.476 | 0.218 | 0.20-0.85 |
| APSIII | 0.668 | 0.687 | 0.629 | 0.541 | 0.759 | 0.605 | 0.217 | 0.20-0.70 |

Supplementary Table 8. Comparison of the proposed model with conventional clinical scoring systems in the internal validation cohort.


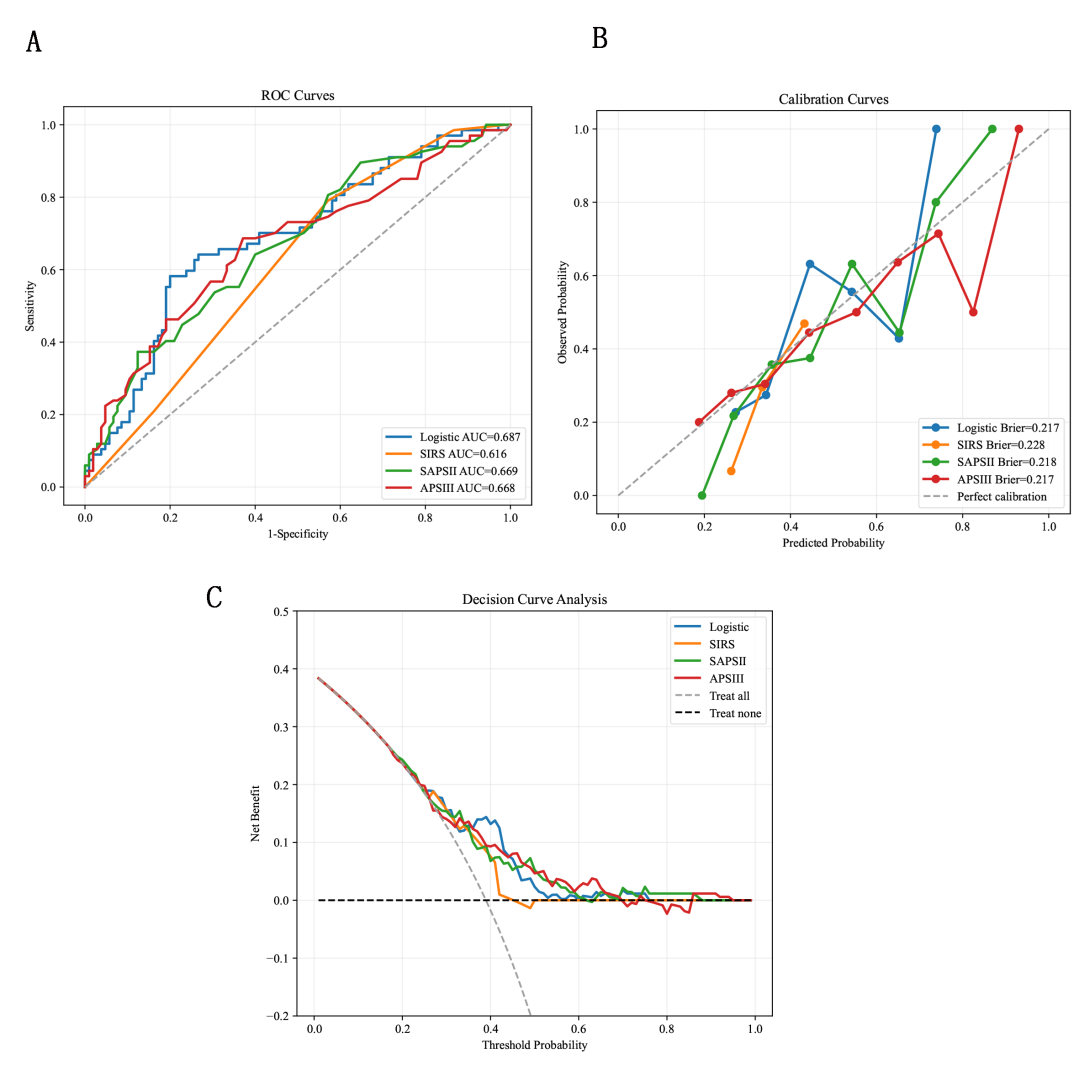


Supplementary Figure 1. Comparison of the proposed logistic regression model with conventional clinical scoring systems: (A) Receiver operating characteristic curves. (B) Calibration curves. (C) Decision curve analysis.
